# Intrinsic Fluorescence–Spin Crossover Interplay in the 3D Fe^II^ Hofmann-Type MOF {Fe^II^(2,6-di(pyridin-4-yl)naphthalene)[M^I^(CN)_2_]_2_}·x(PhNO_2_) (M^I^ = Ag, Au)

**DOI:** 10.1021/acs.inorgchem.6c02744

**Published:** 2026-09-07

**Authors:** Alejandro Orellana-Silla, M. Carmen Muñoz, Gábor Molnár, Carlos Bartual-Murgui, José Antonio Real

**Affiliations:** † Instituto de Ciencia Molecular, Departamento de Química Inorgánica, 201456 Universidad de Valencia, Catedrático José Beltrán 2, Paterna, Valencia 46980, Spain; ‡ Departamento de Física Aplicada, Universitat Politècnica de València, Camino de Vera s/n, Valencia 46022, Spain; § LCC−CNRS, Université de Toulouse, CNRS, Toulouse 31077, France; ∥ Departamento de Química Física, Universitat de València, Dr Moliner 50, Burjassot 46100, Spain

## Abstract

We report the synthesis, characterization, and photophysical properties of two isoreticular 3D Hofmann-type Metal-Organic Frameworks (MOFs), {Fe^II^(2,6-di­(pyridin-4-yl)­naphthalene)­[M^I^(CN)_2_]_2_}·x­(PhNO_2_) (M = Ag (**1Ag**), Au (**1Au**)), incorporating a naphthalene-based fluorescent ligand as bridging pillars. Single-crystal X-ray diffraction analysis of **1Au** reveals a doubly interpenetrated primitive cubic (**pcu**) framework constructed from {Fe^II^_4_[M^I^(CN)_2_]_4_}_n_ grids pillared by the organic linker, with nitrobenzene molecules occupying the accessible voids and engaged in multiple host–guest interactions. Magnetic and calorimetric studies show a thermally induced and complete spin crossover (SCO) in both compounds, with similar transition temperatures but distinct cooperativity, including a multistep behavior pronounced in the Ag derivative. Variable-temperature fluorescence measurements of **1Ag** and **1Au** display an emission band centered at ca. 585 nm, whose intensity decreases upon cooling in correlation with the LS state population. This behavior is attributed to a reabsorption process consistent with an inner filter effect, highlighting the role of the spin-state-dependent optical properties of the framework. Structural analysis suggests that differences in the metal–cyanide linkage influence host–guest interactions and lattice response, thereby modulating the SCO profile. These results provide insights into the interplay between intrinsic fluorescence and SCO in porous frameworks and contribute to the rational design of multifunctional SCO-based materials.

## Graphical abstract

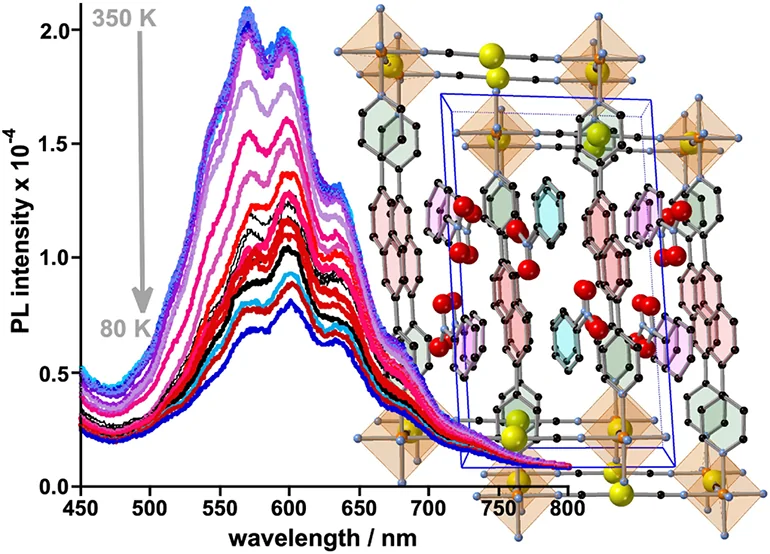

## Introduction

The spin crossover (SCO) phenomenon in Fe^II^ coordination compounds arises from the delicate balance between ligand field strength and electron pairing energy, enabling a reversible switching between low-spin (LS, t_2g_^6^e_g_^0^) and high-spin (HS, t_2g_^4^e_g_^2^) electronic configurations.^1–4^ This interconversion can be triggered by external perturbations such as temperature, pressure, light irradiation or chemical environment, and is typically accompanied by pronounced modifications in the metal–ligand bond distances and overall molecular geometry.^5,6^ In the solid state, these local structural changes can propagate through intermolecular interactions, giving rise to cooperative effects that manifest as abrupt, gradual, multi-step or hysteretic transitions.^7–9^ The strong coupling between electronic configuration and lattice degrees of freedom makes SCO materials particularly appealing as switchable systems with tunable physical properties.

Within this field, Hofmann-type Metal-Organic Frameworks (MOFs) constitute a prominent class of SCO materials owing to their structural versatility and predictable assembly.^10–15^ These frameworks are constructed from Fe^II^ nodes interconnected through cyanometallate linkers, forming extended grids that can be expanded into two- (2D) or three-dimensional (3D) architectures depending on the nature of the axial organic ligand. A general description of this family can be expressed by the formula {Fe^II^(L)_*x*_[M­(CN)_*y*_]_*z*_}, where L represents the axial ligand and M the metal center of the cyanometallate unit. The dimensionality of the resulting framework is largely dictated by the connectivity of L: monodentate ligands (x = 2) typically lead to layered 2D architectures, whereas bis-monodentate bridging ligands (x = 1) promote the formation of 3D networks. The latter often display permanent porosity and, in many cases, interpenetrated frameworks capable of accommodating guest molecules, depending on the nature of the cyanometallate building block. For M = Ag^I^ or Au^I^, linear dicyanometallate units [M^I^(CN)_2_]^−^ (y = 2, z = 2) generate relatively open networks that frequently undergo double interpenetration because of their low framework density, whereas for M = Ni^II^, Pd^II^, or Pt^II^, square-planar tetracyanometallate units [M^II^(CN)_4_]^2–^ (y = 4, z = 1) typically afford more compact architectures with a reduced tendency toward interpenetration. This combination of structural regularity and chemical flexibility enables systematic modification of both the local coordination environment and long-range interactions, offering fine control over the SCO response.

Beyond their magnetic bistability, SCO Hofmann-type MOFs have proven to be excellent candidates for the development of multifunctional materials.^16–18^ In particular, coupling SCO with luminescence has attracted considerable interest as it provides an accessible optical probe of the spin state.^19–21^ Strategies to achieve this include the incorporation of emissive species that are not directly integrated into the SCO framework backbone, such as guest molecules confined within the pores or externally doped luminophores,^22–26^ as well as the use of inherently fluorescent ligands embedded within the SCO framework.^22,27–39^ In both approaches, the interaction between the SCO-active centers and the chromophore units can give rise to a variety of mechanisms, including energy transfer processes and reabsorption effects (e.g., inner filter effect). The resulting emission response is often highly sensitive to subtle structural variations, such as framework interpenetration or host–guest interactions, which can strongly modulate the interplay between spin transition and luminescence. Recently, we demonstrated the latter strategy in a homologous family of Fe^II^ Hofmann-type frameworks incorporating the 1,6-di­(pyridin-4-yl)­pyrene ligand (Scheme 1, left) and dicyanometallate building blocks (M^I^ = Ag, Au), where intrinsic fluorescence was successfully coupled to spin crossover.^39^

**Scheme 1:**
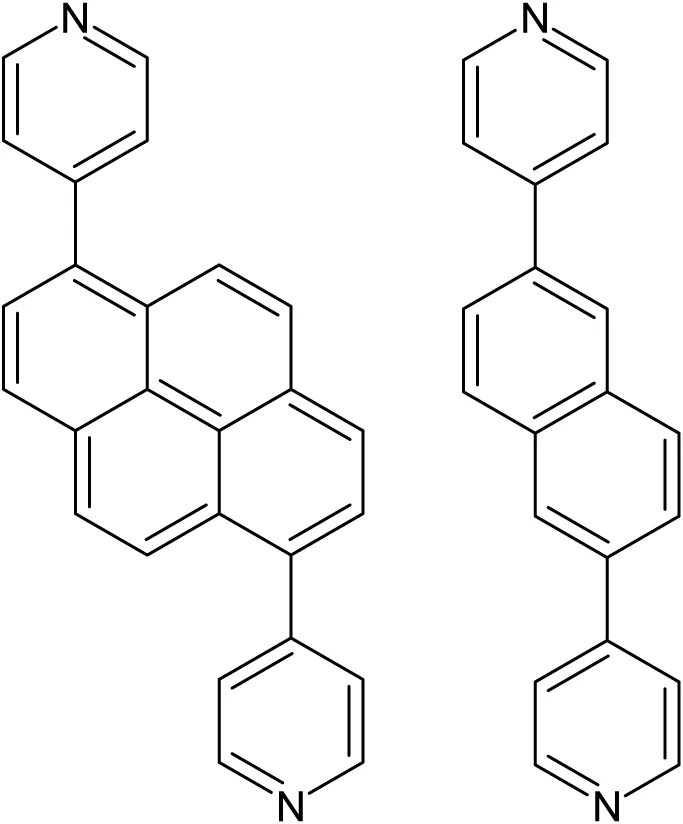
(Left) Structure of the Ligand 1,6-Di­(pyridin-4-yl)­pyrene (Used in Reference 39) and (Right) Ligand 2,6-Di­(pyridin-4-yl)­naphthalene (dpyNaph) Used in This Work

Motivated by these findings, the present work explores the influence of the bridging ligand by replacing the pyrene-based linker with the less bulky 2,6-di­(pyridin-4-yl)­naphthalene ligand (dpyNaph, Scheme 1, right). In this context, we present two Fe^II^ Hofmann-type MOF analogues formulated as {Fe^II^(dpyNaph)­[M^I^(CN)_2_]_2_}·x­(PhNO_2_) (M^I^ = Ag, Au). These compounds form 3D doubly interpenetrated networks with accessible voids occupied by nitrobenzene molecules. Both materials undergo a thermally induced and essentially complete SCO, although with notable differences in their cooperative behavior despite their isoreticular nature. In addition, the frameworks exhibit intrinsic fluorescence arising from the naphthalene-based organic linker, whose intensity evolves with temperature in correlation with the spin transition. Interestingly, the emission decreases upon cooling, pointing to a mechanism governed by reabsorption processes consistent with an inner filter effect. Comparison with the previously reported pyrene-based analogues reveals that the combination of the less bulky bridging ligand and the different guest loading modifies the host–guest interactions and framework flexibility, resulting in markedly different SCO profiles and fluorescence modulation. Likewise, subtle variations in the metal–cyanide linkage between the Ag and Au analogues influence the framework flexibility and the balance between host–host and host–guest interactions, ultimately determining the cooperativity of the spin crossover. These results highlight the intricate interplay between framework structure, guest inclusion and photophysical response in SCO-active materials.

## Results

### Synthesis

Both compounds were synthesized by slow diffusion in H-shaped tubes containing in one side a MeOH:PhNO_2_ (2:1) solution of a 2:1 mixture of K­[M^I^(CN)_2_] (M^I^ = Ag or Au) and dpyNaph and in the other side a MeOH:PhNO_2_ (2:1) solution of [Fe­(H_2_O)_6_]­(BF_4_)_2_ (see Experimental Section). The composition of the yellow crystalline compounds, [Fe­(dpyNaph)­[M^I^(CN)_2_]_2_]·(xPhNO_2_)) [M^I^ = Ag (**1Ag**, n = 1.5), Au (**1Au**, n = 2)], was confirmed by single crystal, thermogravimetric, IR spectroscopy and elemental analyses. The thermogravimetric analysis confirmed that the frameworks include 1.5 (**1Ag**) and 2 (**1Au**) molecules of PhNO_2_ which are released at temperatures above ca. 390 K (Figure S1). The presence of the guest was also confirmed by the presence of two characteristic very intense and sharp symmetric and asymmetric νNO_2_ stretching modes (Figure S2). Only the crystal structure of **1Au** has been fully analyzed due to the lack of quality of the **1Ag** crystals. However, the close similarity between the experimental XRPD patterns (Figure 1) strongly suggests that both compounds, as it is common in this type of compounds, display very similar structures. In this respect, it deserves to be noted that the very strong diffraction at 2θ ca. 5° ([001]) corresponds to the distance mediated between two consecutive 2D [Fe­(Au^I^(CN)_2_)_2_]_n_ layers interconnected by the dpyNaph bridging ligands and the same diffraction is observed, among others, in the Ag^I^ derivative. Obviously, the small differences in position and number of peaks in the PXRD pattern (Figure 1) are reasonably associated with differences in intensity and number of steric constraints produced by the guests in each framework (see Crystal Structure Section).

**Figure 1:**
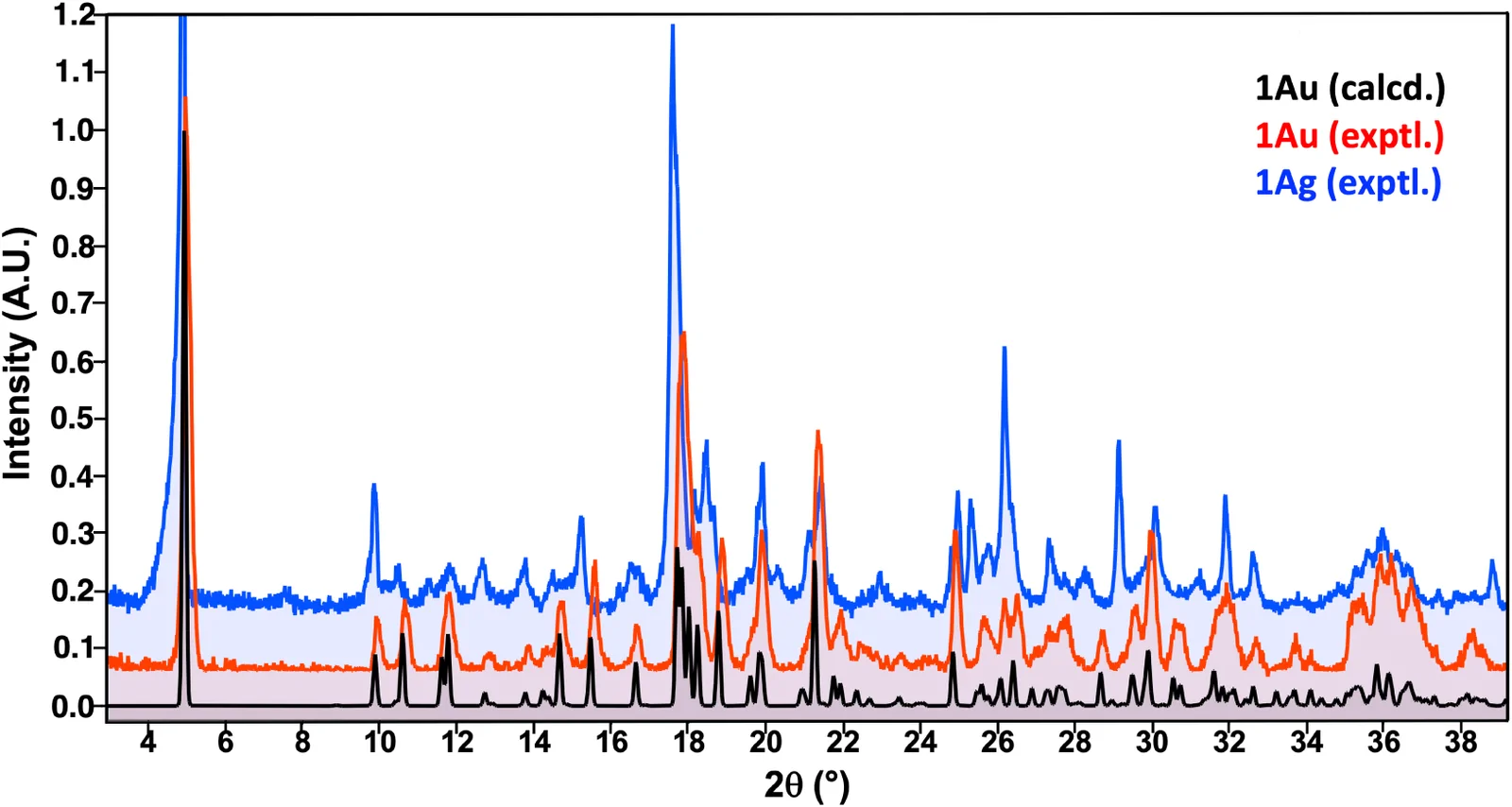
Powder X-ray diffraction patterns for **1Au** (calculated, black) and **1Ag** (experimental, blue)/**1Au** (experimental, red).

### Spin Crossover Behavior

#### Magnetic Measurements

Figure 2 displays the magnetic properties of **1Ag** and **1Au** expressed in the form of the *χ*_*M*_ *T* product recorded at a temperature scan rate of 2 K min^–1^, being *χ*_*M*_ = molar magnetic susceptibility and *T* = temperature (see Experimental Section). At 350 K, the *χ*_*M*_ *T* value, 3.40/3.48 cm^3^ K mol^–1^, are consistent with an essentially fully populated HS state for **1Ag**/**1Au**. Upon cooling, the *χ*_*M*_ *T* value of **1Ag** exhibits a decrease through a two-step process. First, a relatively abrupt drop of *χ*_*M*_ *T* is observed below 320 K, reaching ca. 1.60 cm^3^ K mol^–1^ at 250–255 K. The *χ*_*M*_ *T* decrease of ∼1.8 cm^3^ K mol^–1^ in the first step corresponds to the spin conversion of roughly 50% of the Fe^II^ centers with an associated *T*_1/2_ of ca. 282 K. Below 250–255 K, the slope of the *χ*_*M*_ *T* vs. *T* plot markedly decreases displaying a much more gradual and poorly marked multi-stepped spin conversion, centered at *T*_1/2_ ≈ 180 K, that is nearly complete at 100 K (χ_M_T ≈ 0.13 cm^3^ K mol^–1^). In contrast, the *χ*_*M*_ *T* value for **1Au** remains essentially constant in the temperature range 350–290 K, but below 290 K, it undergoes a relatively cooperative decrease from 3.40 to 0.60 cm^3^ K mol^–1^, consistent with an almost complete SCO behavior centered at *T*_1/2_ = 268 K. Although this *χ*_*M*_ *T* drop accounts for the spin transition of most Fe^II^ centers (∼92%), a second step involving the remaining ∼8% is observed at *T*_1/2_ = 215 K.

**Figure 2:**
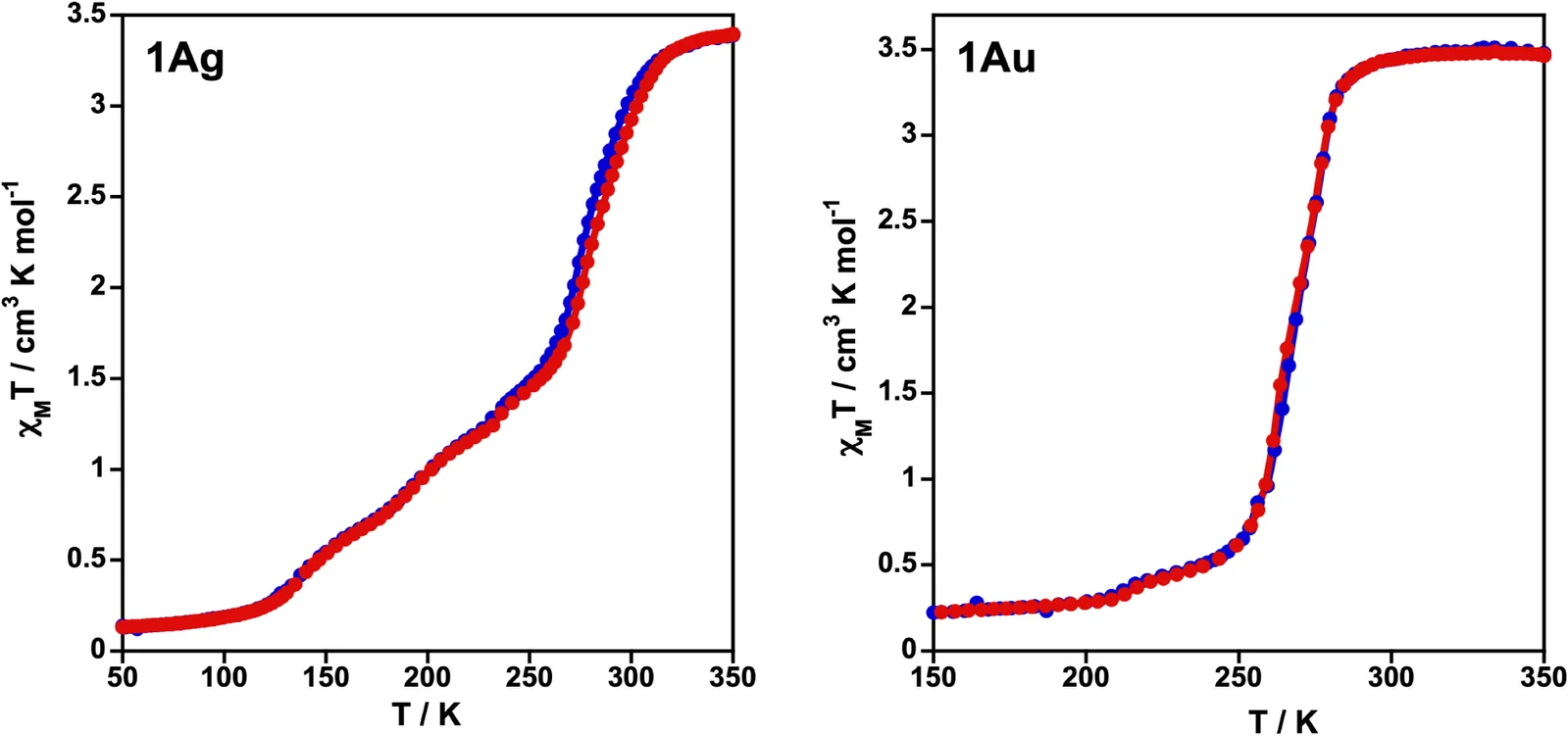
*χ_M_T* vs. T plots for **1Ag** and **1Au** (filled blue and red circles correspond to the cooling and heating modes, respectively).

#### Calorimetric Measurements

The thermal dependence of the molar heat capacity associated with the SCO, *ΔC*_*p*_, has been obtained from differential scanning calorimetry (DSC) measured at 5 K min^–1^ for both derivatives (Figure 3). Consistently with the magnetic data, the corresponding *ΔC*_*p*_ vs T profiles denote the occurrence of a multi-stepped SCO. The average enthalpy and entropy variations associated with the SCO are ΔH = 18.4/21.4 kJ mol^–1^ and ΔS = 69/81 J K^–1^ mol^–1^ for **1Ag**/**1Au**. The corresponding *T*_1/2_ = ΔH/ΔS values obtained are 274, 192, and 138 K for **1Ag** and 269 and 213 K for **1Au**, respectively. These values agree reasonably well with those obtained from magnetic data.

**Figure 3:**
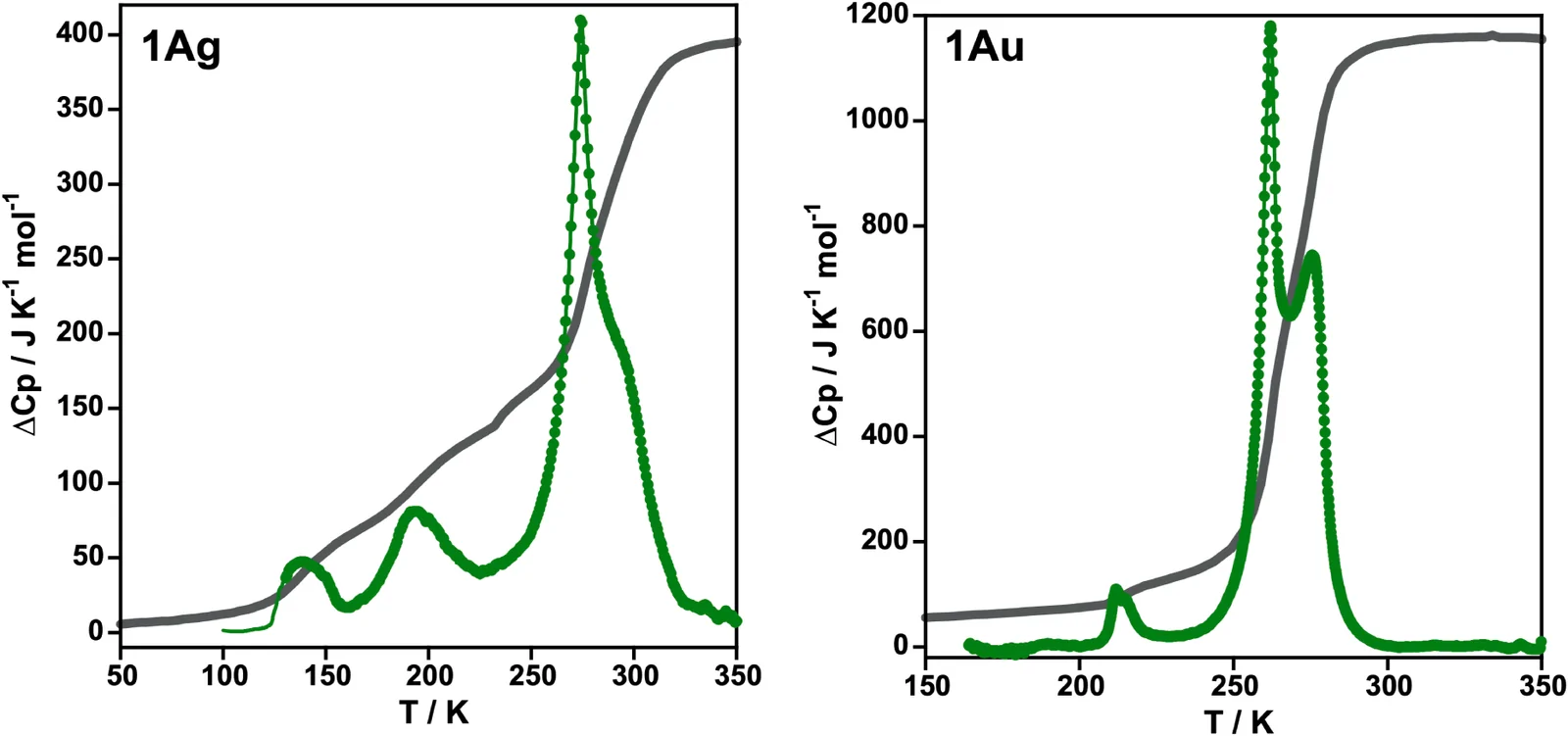
*ΔC_p_* vs. *T* plots for **1Ag** and **1Au** (green) (the thin line in the 130–100 K range corresponds to extrapolation of the data). The underlying gray line correspond to the *χ_M_T* vs. *T* plot.

### Crystal Structure

The crystal structure of **1Au** has been analyzed via single crystal X-ray diffraction at 120 and 315 K where, according to the magnetic data, the complex is in the LS and HS state, respectively. Whatever the temperature, the crystal adopts the triclinic *P-1* space group. Relevant crystallographic data are gathered in Table S1. Table 1 summarizes relevant bond lengths and angles concerning the [Fe^II^N_6_] and [Au^I^C_2_] sites. Figure 4a displays a key fragment of the structure emphasizing the corresponding atom numbering. The Fe^II^ centers are located at the middle of a slightly elongated octahedron with the four equatorial positions occupied by two crystallographically distinct almost linear [Au^I^(CN)_2_]^−^ groups, while the axial positions are occupied by two dpyNaph ligands. The average <Fe–N> bond length at 120 and 315 K, 1.955 and 2.162 Å, are consistent with the expected for the Fe^II^ LS and HS states, respectively. Furthermore, the bond length and octahedral volume (V_[FeN6]_) variations, 0.207 Å and 3.52 Å^3^, corresponds to a complete HS-LS transformation in agreement with the magnetic data. Surprisingly, the angular distortion parameter of the [FeN_6_] from the ideal octahedron, defined as the sum of the angular deviation of the 12 *cis* angles (Σ = Σ_1_^12^[*α*_*i*_ – 90]),^40–42^ is very similar for the HS and LS states. The same is observed for the trigonal distortion value, *Θ* = Σ_1_^24^(|60 – *φ*_i_|), *φ*_i_ being the angles generated by superposition of two opposite faces of the octahedron, which are virtually identical (see Figure S3).

**Table 1:** Bond Lengths and Angles of the Fe^II^ and Au^I^ Coordination Centers for **1Au**

Fe–N_i_/ Å	120 K	315 K
Fe1–N1	1.936(4)	2.140(9)
Fe1–N2	1.931(3)	2.127(8)
Fe1–N3	1.924(4)	2.150(8)
Fe1–N4	1.932(3)	2.146(8)
Fe1–N5	2.001(3)	2.206(7)
Fe1–N6	2.004(4)	2.195(7)
<Fe–N>	1.955	2.161
V_[FeN6]_/Å^3^	9.951	13.470
Σ/°	10.3	8.4
Θ/°	23.6	24
Au1–C1	1.985(4)	1.967(12)
Au2–C2	1.980(4)	1.972(10)
Au2–C3	1.981(4)	1.967(12)
Au1–C4	1.984(4)	1.996(8)
Au1···Au2	3.0876(2)	3.1338(6)
C(1)–Au(1)–C(3)	178.1(2)	177.1(4)
C(2)–Au(2)–C(4)	177.9(2)	177.8(3)

**Figure 4:**
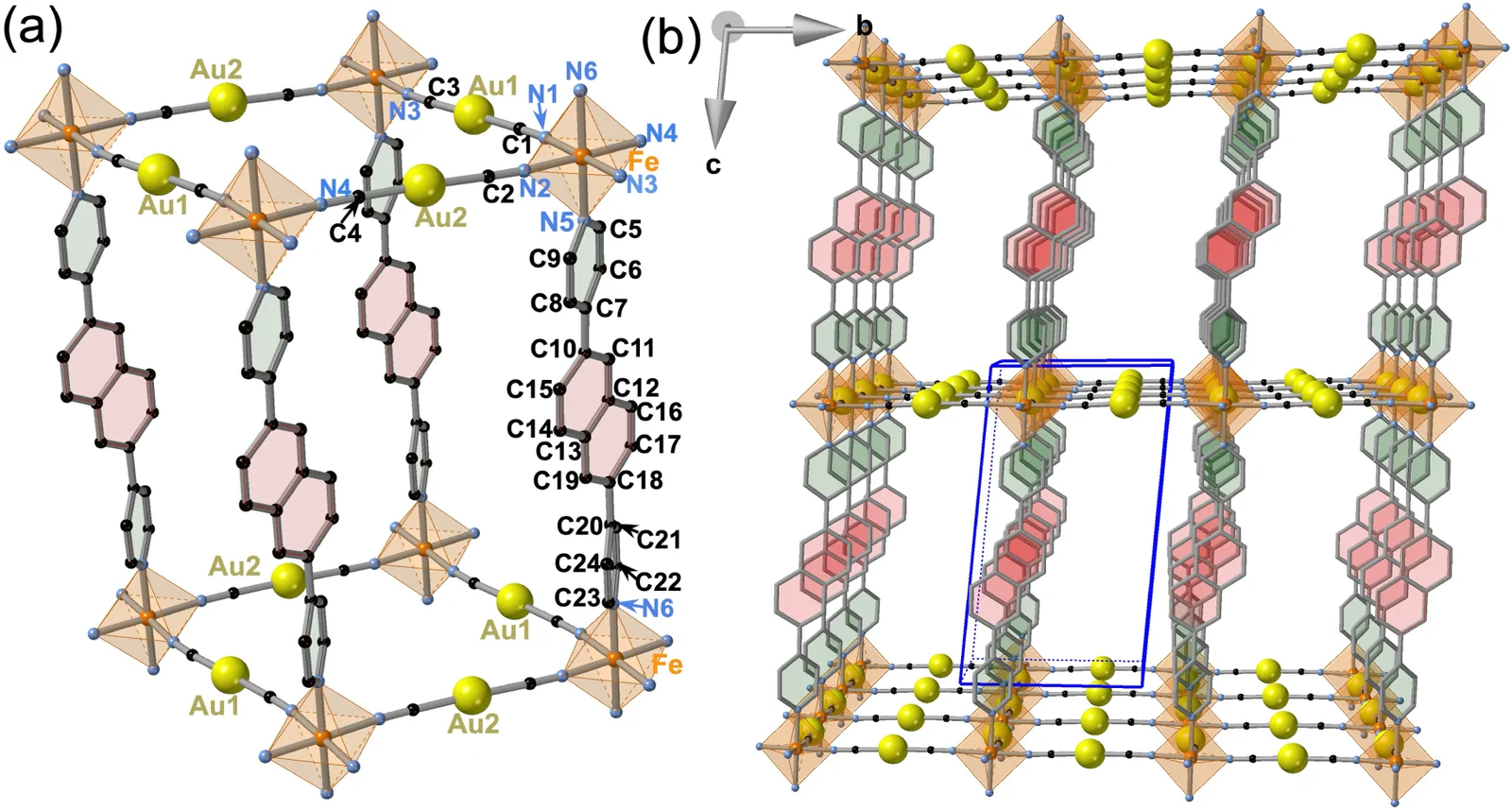
Characteristic structural motive of the framework emphasizing the coordination of the Fe^II^ centers and atom numbering (a). Extended fragment of one of the two interpenetrated frameworks (b).

The [Au­(CN)_2_]^−^ groups act as bridging ligands, defining almost planar layers composed of {Fe_4_[Au­(CN)_2_]_4_} square tiles (see Figure 4a), which share Fe­(II) nodes and [Au­(CN)_2_]^−^ edges to generate the infinite two-dimensional network. These layers are stacked perpendicular to the *c* direction and are separated by 17.91 and 17.49 Å at 315 and 120 K, respectively. The layers are connected through the bridging dpyNaph pillar ligands, thereby affording a three-dimensional framework with **pcu** topology (Figure 4b). The open nature of the structure favors the interpenetration of a second identical framework, both locked each other through Au···Au interactions (Figure 5a, Table 1). Despite the interpenetration of the two frameworks there is room to dock two crystallographically distinct molecules of PhNO_2_ (Figure 5b). In the LS state at 120 K, the PhNO_2_ guest molecules are in such a way that display mutual strong intermolecular interaction through the NO_2_ groups (O4···N7) (see Table S2 and Figure S4). Furthermore, the PhNO_2_ molecules are also involved in short intermolecular contacts with the dpyNaph pillars via offset π···π stacking, (C26···C16, C36···C13). In addition, the nitro function interacts with the central naphthalene moieties (O4···H11–C11, O3···H16–C16) and the peripheral pyridine ring (O4···H6–C6, O2···H8–C8). As expected, at 315 K where the compound is HS, these interactions are much more moderated (Table S3, Figure S5).

**Figure 5:**
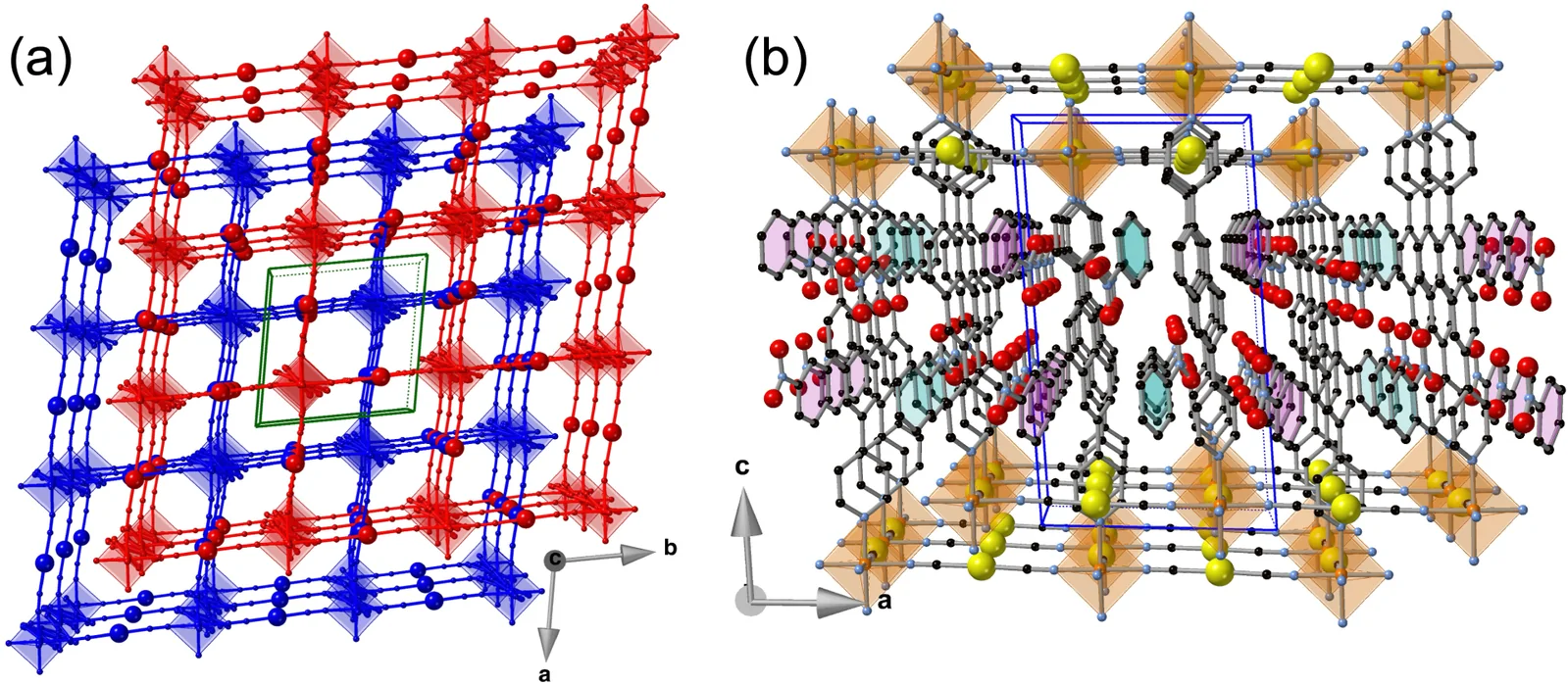
Two perpendicular views of the interpenetrated frameworks without (a) and with (b) the two crystallographically distinct guest PhNO_2_ molecules colored in pink and blue colors.

### Fluorescent Properties

Figure 6 displays the temperature dependence of the emission spectra after excitation at 365 nm of compound **1Ag** and **1Au**. The emission spectra of both complexes are similar and consist of a wide band centered at ca. 585 nm featuring a vibronic fine structure. Upon cooling from 323 K down to 183 K, the emission spectra intensity decreases 51% and 24% for **1Ag** and **1Au**, respectively. Contrarily to what usually is observed, the fluorescence emission upon cooling decreases in a parallel fashion to the increase of population of the Fe^II^ LS centers (Figure 7). This variation is consistent with an inner filter effect, in which the emitted photons are more efficiently reabsorbed in the LS state, leading to a reduction of the observed emission intensity. This interpretation is supported by the color change exhibited by **1Ag** and **1Au** from pale yellow in the HS state to dark red in the LS state, a characteristic feature of Hofmann-type Fe^II^ SCO compounds. It is well established that, in absence of strong MLCT absorption bands, the weak color of the HS state originates from the spin-allowed but weak ^5^T_2_ → ^5^E transition located in the 800–1000 nm region, whereas the intense color of the LS state arises from the much stronger ^1^A_1_ → ^1^T_1_ and ^1^T_2_ d-d absorption bands spanning approximately the 330–670 nm region.^43^ As reported for other fluorescent Fe^II^ spin crossover materials, the HS-to-LS transition therefore produces a marked increase in absorption within the same spectral window where the fluorescence emission takes place, making an inner filter effect a plausible explanation for the observed luminescence attenuation. Consistently, the decrease in luminescence occurs precisely within the temperature range where the spin crossover takes place, whereas organic fluorophores are generally expected to display an increase in emission intensity upon cooling. Further support for this hypothesis is provided by the fluorescence lifetime measurements of compound **1Au**, which remain essentially unchanged across the spin crossover transition despite the pronounced decrease in emission intensity (Figure S6). Such behavior is consistent with an inner filter mechanism, which attenuates the detected emission without significantly affecting the intrinsic excited-state lifetime of the fluorophore.

**Figure 6:**
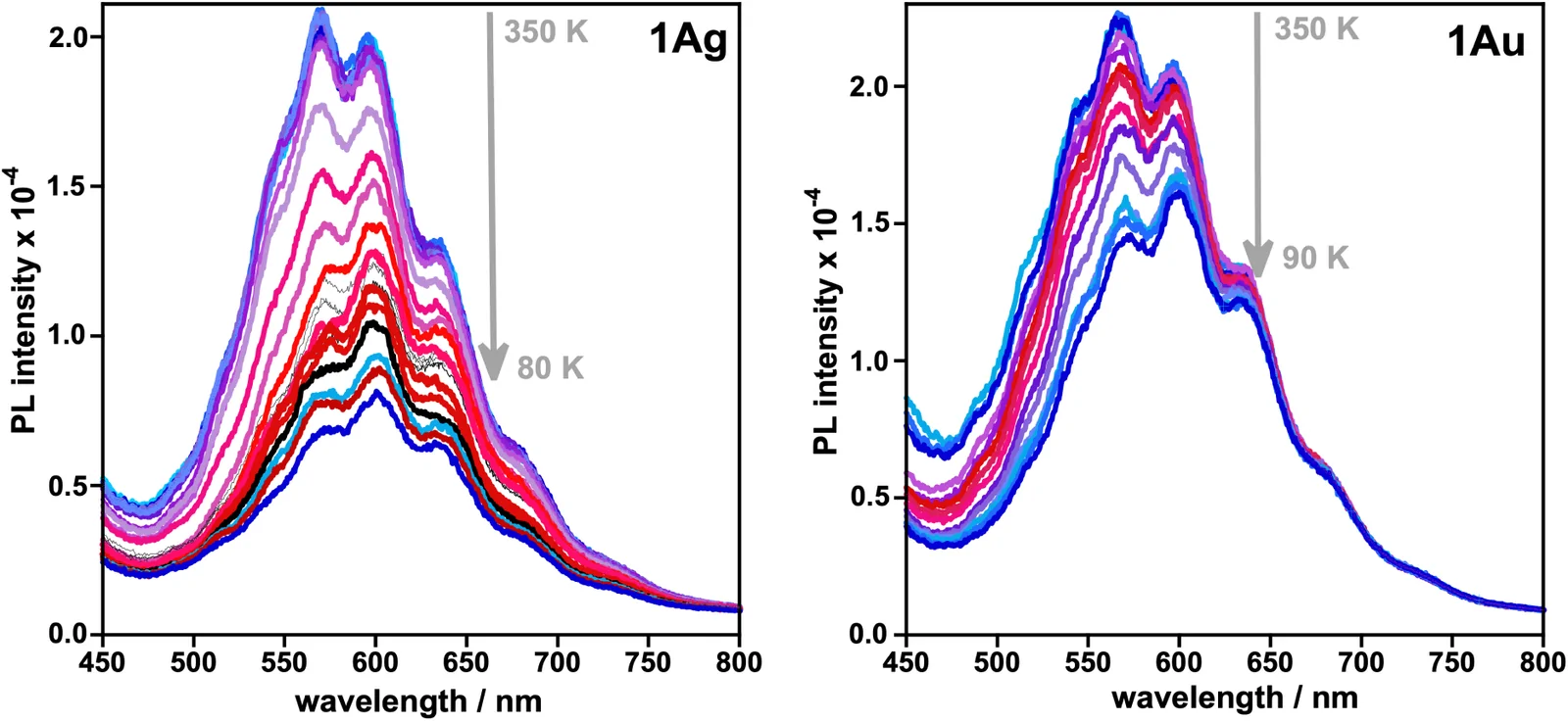
Temperature dependence of the emission spectra after excitation at 365 nm of compound **1Ag** and **1Au**.

**Figure 7:**
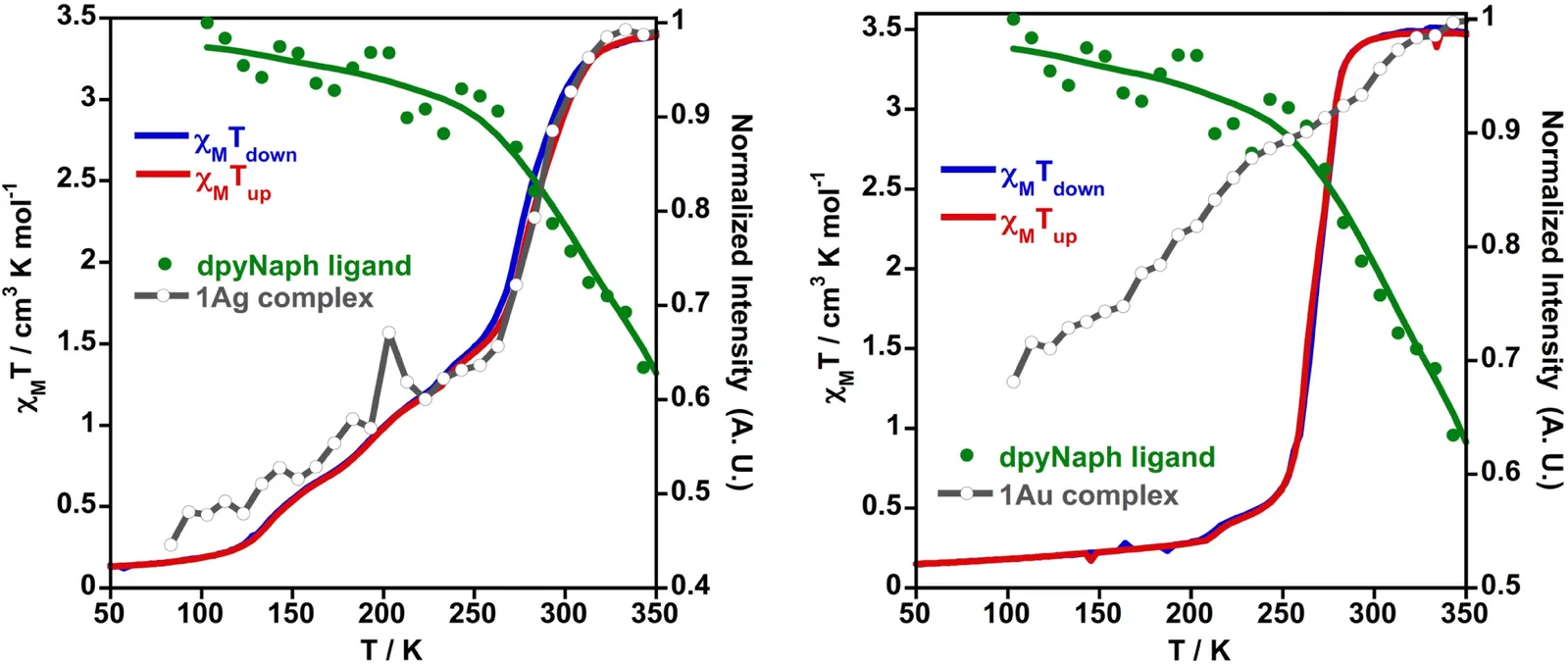
Temperature dependence of the integrated photoluminescence intensity (excited at 365 nm) for **1Ag** and **1Au** (gray open circles) and for the free dpyNaph ligand (green filled dots). Lines are guides for the eyes. For comparison, the temperature dependence of the *χ_M_T* product is also shown (red/blue lines).

For comparison, the thermal evolution of the photoluminescence of the free ligand dpyNaph was also investigated (Figure S7). In this case, the emission spectra display a broader band than those of the complexes, spanning a wider spectral range toward shorter wavelengths (425–700 nm versus 500–700 nm for the clathrates) and centered at 540 nm. Moreover, in contrast to the behavior observed for **1Ag** and **1Au**, the emission intensity increases upon cooling (see green line in Figure 7), as expected from the progressive suppression of non-radiative decay pathways at lower temperatures.

## Discussion

In the search for new synergies between SCO and fluorescence we have investigated two nitrobenzene clathrates of the Hofmann-type Fe^II^ MOFs, **1Ag** and **1Au**, assembled from dpyNaph bridging ligands and dicyanometallate [M^I^(CN)_2_]^−^ (M^I^ = Ag, Au) building blocks. The thermal dependence of the magnetic and calorimetric properties reveals a multi-step SCO behavior, which is especially pronounced for **1Ag**. Although both compounds display very similar *T*_1/2_ values, their SCO profiles diverge significantly below 260 K despite the isoreticular nature of both frameworks. In **1Au**, the SCO occurs practically in one steep step accompanied by very subtle changes in slope, thereby suggesting a predominance of moderately strong host–host and host-guest elastic interactions favoring a more cooperative behavior. In contrast, for **1Ag**, the drastic change in slope of the SCO profile after ca. 50% of HS-to-LS spin state conversion makes it much less cooperative thus reflecting a predominance of weaker interactions. This marked difference may be related to the different flexibility featuring the [M^I^(CN)_2_]^−^ building blocks combined with the effect of host-guest interactions during the contraction-expansion of the frameworks upon SCO. In **1Au**, these interactions promote a certain degree of cooperativity, leading to a relatively abrupt SCO behavior that can be associated with the more rigid and linear nature of the [Au­(CN)_2_]^−^ building block. In contrast, the greater tendency of the [Ag­(CN)_2_]^−^ bridge to deviate from linearity, and consequently to distort the framework acting as some kind of shock absorber,^6,44,45^ may explain both the splitting of the ν_C≡N_ stretching mode into three components in **1Ag** (Figure S2) and the partial disruption of the communication between spin-switching centers, ultimately leading to reduced cooperativity. Konar et al. have recently reported the magnetic properties and crystal structure of the homologous {Fe^II^(dpyNaph)­[Ag^I^(CN)_2_]_2_}·{3CHCl_3_} clathrate.^46^ Despite the latter compound crystallizes in the monoclinic P2_1_/c space group, it shows a very similar structure as the title compounds **1Ag** and **1Au**, characterized by the first diffraction peak at 5.025° (17.625 Å) which corresponds to the separation between two consecutive {Fe_4_[Ag­(CN)_2_]_4_}_n_ layers linked by the dpyNaph ligands (see Figure S8). The CHCl_3_ solvate displays a mainly gradual multi-step SCO taking place in the 270–150 K temperature range featuring a *T*_1/2_ ≈ 206 K, a value ca. 60 K smaller than that observed for **1Ag**. Obviously, the number and nature of the SCO steps, as well as the temperatures at which they occur, depend not only on the identity and amount of guest molecules (CHCl_3_ vs PhNO_2_), but also on the delicate balance between host–guest, guest–guest, and host–host supramolecular interactions. This has been recently shown by Tong et al. on the also closely related Hofmann-type SCO complex Fe­(1,4-dpyNaph)­[Ag­(CN)_2_]_2_·1Guest being 1,4-dpyNaph = 1,4-di­(pyridin-4-yl)-naphthalene and Guest = Ph_2_S (diphenylsulfide), Ph_2_SO (diphenylsulfoxide) and Ph_2_SO_2_ (diphenylsulfone).^47^ Similarly, the structure is defined by the interpenetration of two identical **pcu** frameworks that differ essentially in the position of the pyridine donors on the naphthalene rings and in a more marked undulation of the [Fe_4_(Ag­(CN)_2_)_4_]_n_ grids that apparently increases with the size of the guest. The completeness of the SCO profile clearly depends on the size of the guest and, as the authors state, on the adaptability of the guest to the structural changes of the host derived from the SCO. The Ph_2_S clathrate displays a complete four step transition with characteristic temperatures 194, 177, 124, 108 K, while the Ph_2_SO clathrate undergoes an incomplete two-step centered at 233 and 140 K, and the Ph_2_SO_2_ clathrate shows a strongly incomplete and gradual SCO behavior below 120 K. Interestingly the Ph_2_S clathrate display a broad fluorescent emission centered at 523 nm at 300 K that undergoes a blue shift to 505 nm when cooling at 150 K. The intensity of the emission displays ca. 20% attenuation because of the HS-to-LS transformation involving 50% of the Fe^II^ centers, however, unexpectedly, upon further cooling to reach 100% of the LS state, the intensity markedly increases ca. 40% in the temperature window 150–100 K.

Coming back to the PhNO_2_ clathrates in **1Ag** and **1Au**, they show characteristic SCO temperatures markedly higher than that of the CHCl_3_ solvate and those of the Ph_2_S, Ph_2_SO and Ph_2_SO_2_ ones in the 1,4-dpyNaph framework. In any case, this difference cannot be attributed to the small structural differences of the organic pillars, but rather to specific guest–guest and host-guest interactions arising from the greater steric demand of the guest molecules, particularly in the latter case, where the two phenyl rings linked through the sulfur/sulfoxide/sulfone moieties define a mutual angle of ca. 109°. In contrast, as shown for **1Au**, the two crystallographically distinct PhNO_2_ molecules are accommodated in a parallel arrangement with respect to the aromatic rings of the dpyNaph pillars and therefore require significantly less space between the doubly interpenetrated frameworks. Concerning the above mentioned CHCl_3_ clathrate, the three guest molecules are involved in short contacts with the host framework via Cl···π interactions with the pyridine and naphthalene moieties of the dpyNaph ligand and via Cl···Ag interactions.

A comparison with our recently reported homologous Hofmann-type Fe^II^ frameworks based on the larger 1,6-dipyridylpyrene ligand and [M­(CN)_2_]^−^ (M = Ag, Au)^39^ is also instructive. Although both families combine intrinsic fluorescence with thermally induced spin crossover and display a clear correlation between the evolution of the luminescence intensity and the spin state, important differences are observed in their magnetic behavior. The pyrene derivatives undergo well-defined two-step SCO transitions for both Ag and Au analogues, with characteristic transition temperatures lying in a similar temperature range (ca. 190–260 K) to those of the present nitrobenzene clathrates. The main difference therefore lies not in the transition temperatures themselves, but in the shape of the SCO profiles: whereas the pyrene analogues display two clearly resolved steps, the present compounds exhibit a much more gradual multistep transition for **1Ag** and an almost single-step cooperative transition for **1Au**. These differences most likely arise from the combined influence of the different aromatic spacers and, more importantly, the identity, number and supramolecular arrangement of the guest molecules. In the pyrene compounds, one toluene molecule per Fe^II^ center occupies the pores, whereas the present dpyNaph frameworks accommodate approximately 1.5 (**1Ag**) and 2 (**1Au**) nitrobenzene molecules per Fe^II^ center, leading to a different balance of host–guest and guest–guest interactions that governs the cooperativity of the spin transition. Despite these structural differences, both families exhibit the same general photophysical behavior: the fluorescence intensity decreases upon the HS-to-LS conversion while the fluorescence lifetime remains essentially unchanged. These observations consistently support an inner filter (emission–reabsorption) mechanism as the most plausible origin of the luminescence modulation in these luminescent Hofmann-type SCO frameworks.

As far as the SCO–fluorescence synergy is concerned, the intensity of the photoluminescence is clearly modulated by the spin crossover in both compounds, decreasing as the low-spin population increases upon cooling. As discussed above, this behavior is consistent with an inner filter effect arising from a more efficient reabsorption of the emitted photons in the LS state. This interpretation is further supported by the temperature-independent fluorescence lifetime measured for compound **1Au** (Figure S6), which indicates that the attenuation of the emission intensity is not accompanied by significant changes in the intrinsic excited-state decay.

For the **1Ag** derivative, the luminescence attenuation reaches ca. 56% over the temperature interval 350–100 K, whereas for **1Au** the corresponding decrease is only ca. 32%. Although the origin of this quantitative difference remains uncertain, one possible contributing factor could be a different extent of constructive or destructive interference between the ligand-centered emission and emissive states associated with argentophilic or aurophilic interactions, which are known to become more relevant at low temperatures as the M···M distance decrease.^48–51^ However, further spectroscopic and theoretical studies would be required to evaluate the possible contribution of these effects.

## Conclusions

Overall, this work demonstrates how subtle variations in framework flexibility and host–guest interactions can profoundly influence the cooperative spin crossover behavior of closely related Hofmann-type MOFs. Despite their close structural nature, the Ag and Au derivatives exhibit markedly different SCO profiles, highlighting the key role played by the distinct structural adaptability of the [M^I^(CN)_2_]^−^ linkers during the lattice contraction accompanying the HS↔LS transformation. At the same time, both materials display an uncommon fluorescence response in which the emission intensity decreases upon cooling in parallel with the growth of the LS state population, a behavior consistent with a spin-state-dependent inner filter effect. These results underline the intimate relationship between framework topology, guest accommodation and photophysical response in porous SCO materials, and further support the use of intrinsically emissive Hofmann-type networks as versatile platforms for the rational design of multifunctional switchable materials combining magnetic and optical functionalities.

## Experimental Section

### Materials and Reagents

Iron­(II) tetrafluoroborate hexahydrate, potassium dicyanoargentate­(I) and potassium dicyanoaurate­(I) were obtained from commercial sources and used without purification. 2,6-di­(pyridine-4-yl)­naphthalene (dpyNaph) was synthesized as described in the literature.^52^

### Synthesis of **1Ag** and **1Au**

Compounds **1Ag** and **1Au** were prepared with yields 70–80% by liquid–liquid slow diffusion in H-shaped tubes. 1 mL of a solution of MeOH:PhNO_2_ (8:2) containing 0.1 mmol of [Fe­(H_2_O)_6_]­(BF_4_)_2_ was introduced in one side of the tubes, while 1 mL of a solution of MeOH:PhNO_2_ (8:2) containing 0.1 mmol of the ligand dpyNaph and 0.2 mmol of K­[Au­(CN)_2_] was added to the remaining side. The tubes were filled with a solution of MeOH:PhNO_2_ (8:2), sealed with parafilm and left undisturbed for, at least, 2 weeks at room temperature. Elemental analysis for **1Ag** and **1Au**: Calculated for **1Ag** [C_33_H_21.5_Ag_2_FeN_7.5_O_3_ (%)]: C 47.13; H 2.56; N 12.50. Found (%): C 46.81; H 2.59; N 12.42. Calculated for **1Au** [C_36_H_24_Au_2_FeN_8_O_4_ (%)]: C 39.95; H 2.23; N 10.35. Found (%): C 39.78; H 2.27; N 10.31.

### Physical Methods of Characterization

#### Magnetic Measurements

Variable temperature magnetic susceptibility data were recorded with a Quantum Design MPMS2 SQUID magnetometer equipped with a 7 T magnet, operating at 1 T and at temperatures of 1.8–400 K. Experimental susceptibilities were corrected for diamagnetism of the constituent atoms by the use of Pascal’s constants.

#### Calorimetric and Thermogravimetric Measurements

Differential scanning calorimetry measurements were performed using a Mettler Toledo, Model DSC 821e calorimeter. Low temperatures were obtained with an aluminum block attached to the sample holder, refrigerated with a flow of liquid nitrogen, and stabilized at a temperature of 110 K. The sample holder was kept in a drybox under a flow of dry nitrogen gas to avoid water condensation. The measurements were performed using crystalline samples (∼10 mg) of **1Ag** and **1Au** sealed in aluminum pans with a mechanical crimp. Temperature and heat flow calibrations were made with standard samples of indium by using its melting transition (429.6 K, 28.45 J g^–1^). An overall accuracy of ±0.2 K in temperature and ±2% in the heat capacity is estimated. The uncertainty increases for the determination of the anomalous enthalpy and entropy due to the subtraction of an unknown baseline.

#### Elemental Analyses

C, H and N analyses were performed on a CE Instruments EA 1110 CHNS Elemental analyzer.

#### Fluorescence Measurements

Variable temperature luminescence emission spectroscopy measurements have been carried out using an Olympus BX51 microscope coupled to a Princeton Instruments Fergie spectrograph, equipped with a back-illuminated CCD sensor, a 50 μm slit and a 295 mm^–1^ grating blazed at 575 nm. Fluorescence was excited at 365 nm (full width at half maximum, FWHM = 9 nm) using a Thorlabs light emitting diode (LED, M365L3). A high-pass filter (>430 nm) was placed before the entrance slit of the spectrograph to block the photons coming from the LED. Temperature control was achieved using a THMS-600 liquid nitrogen cryostate (Linkam Scientific). Room temperature luminescence lifetime measurements were performed using the time-correlated single photon counting (TCSPC) technique by means of a DeltaFlex (Horiba) instrument equipped with a 303 nm LED (pulse duration 1.2 ns) and a PPD-650 photon counting detector. The emission monochromator wavelength was set to 565 nm and a high-pass filter (>500 nm) was placed before the entrance slit of the monochromator to block the photons coming from the excitation light source. Fittings and lifetime calculations were performed using DAS6 fluorescence decay analysis software (Horiba).

#### Single Crystal X-ray Diffraction

Single-crystal X-ray data were collected on a Nonius Kappa-CCD single crystal diffractometer using graphite monochromated Mo K_α_ radiation (*λ* = 0.71073 Å). A multi-scan absorption correction was performed. The structures were solved by direct methods using SHELXS-2014 and refined by full-matrix least squares on *F* ^2^ using SHELXL-2014.^53^ Non-hydrogen atoms were refined anisotropically and hydrogen atoms were placed in calculated positions refined using idealized geometries (riding model) and assigned fixed isotropic displacement parameters. CCDC files 2555615 and 2555616 contain the supplementary crystallographic data for this article. These data can be obtained free of charge from The Cambridge Crystallographic Data Centre via www.ccdc.cam.ac.uk/ data_request/cif.

#### IR Spectra

IR spectra were recorded using KBr discs in the range of 4000–400 cm^–1^ with a Nicolet iS50R FT-IR spectrometer.

## Supplementary Material



## Notes

Thermogravimetric analyses (Figure S1), infrared spectra (Figure S2), single crystal data (Table S1), overlay of the [FeN6] octahedra in the LS (blue, 120 K) and HS (red, 315 K) (Figure S3), short intermolecular contacts at 120/315 K (Tables S2 and S3 and Figures S4 and S5), lifetime thermal dependence (Figure S6), thermal dependence of the fluorescence spectrum of the free ligand dpyNaph (Figure S7), comparison of the powder X-ray diffraction patterns of the PhNO_2_ clathrates (**1Ag**, **1Au**) and the previously reported dicyanoargentate CHCl_3_ clathrate (Figure S8) (PDF)
