# Supplementary material for: Intrinsic Fluorescence–Spin Crossover Interplay in the 3D FeII Hofmann-Type MOF {FeII(2,6-di(pyridin-4-yl)naphthalene)[MI(CN)2]2}·x(PhNO2) (MI = Ag, Au)
Source: Inorg Chem. 2026 Sep 7;65(37):21639–48. doi: 10.1021/acs.inorgchem.6c02744 (PMC13599387; doi:10.1021/acs.inorgchem.6c02744)

Supporting Information

Intrinsic Fluorescence – Spin Crossover Interplay in the 3D Fe^{II} Hofmann-type MOF $\{Fe^{II}$(2,6-di(pyridin-4-yl)naphthalene)$[M^{I}(CN)_2]_2\}\cdot x(PhNO_2)$ (M^{I} = Ag, Au)

Alejandro Orellana-Silla,[a] M. Carmen Muñoz,[b] Gábor Molnár,[c] Carlos Bartual-Murgui,*[d] José Antonio Real*[a]

[a]Instituto de Ciencia Molecular, Departamento de Química Inorgánica, Universidad de Valencia, Catedrático José Beltrán 2, 46980 Paterna, Valencia, Spain.
[b] Departamento de Física Aplicada, Universitat Politècnica de València, Camino de Vera s/n, 46022, Valencia, Spain.
[c]LCC-CNRS, Université de Toulouse, CNRS, 31077 Toulouse, France.
[d] Departamento de Química Física, Universitat de València, Dr Moliner 50, Burjassot, 46100, Spain
Corresponding Authors Email addresses: jose.a.real@uv.es; carlos.bartual@uv.es

page

Figure S1. Thermo-gravimetric analysis for **1Ag** and **1Au**..**S2**

Figure S2. Infrared spectra of **1Ag** and **1Au** emphasizing the presence of the characteristic NO_2 and C≡N stretching modes... **S2**

Table S1. Single crystal crystallographic data for **1Au** at 120 and 315 K.....................................**S3**

Figure S3. Overlay of the $[FeN_6]$ octahedrons in the LS (blue, 120 K) and HS (red, 315 K) emphasizing the small angular distortion induced by the change of spin state..........................**S4**

Table S2. Short intermolecular contacts smaller than the sum of the Van der Waals radii (VdW) for **1Au** at 120 K...**S4**

Figure S4. Intermolecular contacts shorter than the sum of the Van der Waals radii (VDW) for **1Au** at 120 K. Bicolor rods display the contacts indicated in **Table S2**...**S5**

Table S3. Short intermolecular contacts smaller than the sum of the Van der Waals radii (VdW) for **1Au** at 315 K...**S6**

Figure S5. Intermolecular contacts shorter than the sum of the Van der Waals radii (VDW) for **1Au** at 315 K. Bicolor rods display the contacts indicated in **Table S3**.. **S6**

Figure S6. Intensity average lifetime as a function of temperature for **1Au**................................**S7**

Figure S7. Thermal dependence of the fluorescence spectrum of the free ligand dpyNaph......**S7**

Figure S8. Comparison of the lowest 2θ diffraction peaks belonging to the 001 reflection for the title compounds **1Ag** (black line) and **1Au** (red line) compared with the homologous 100 peak of the **$3CHCl_3$** solvate (see text)...**S8**

Figure S1. Thermo-gravimetric analysis for **1Ag** and **1Au**.

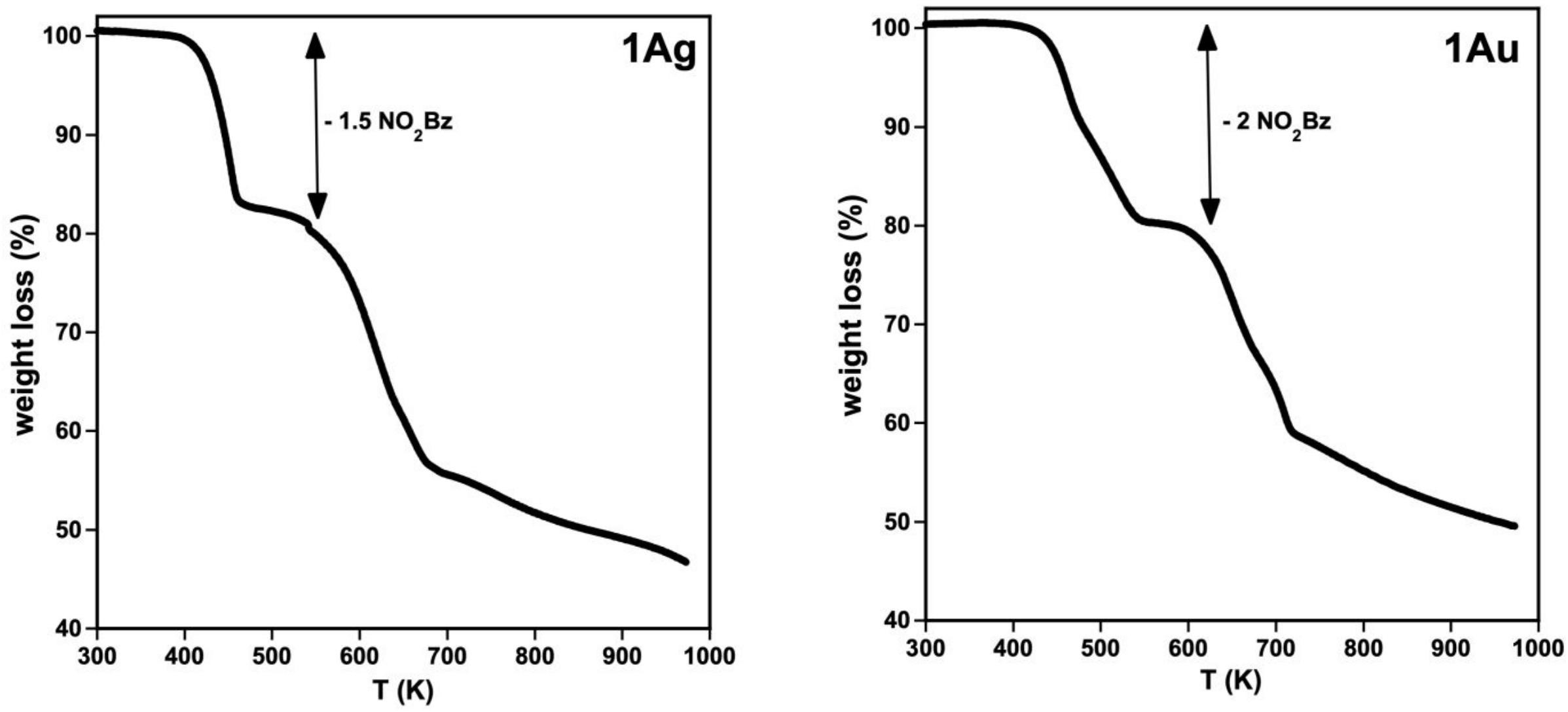

Figure S2. Infrared spectra of **1Ag** and **1Au** emphasizing the presence of the characteristic NO_2 and C≡N stretching modes.

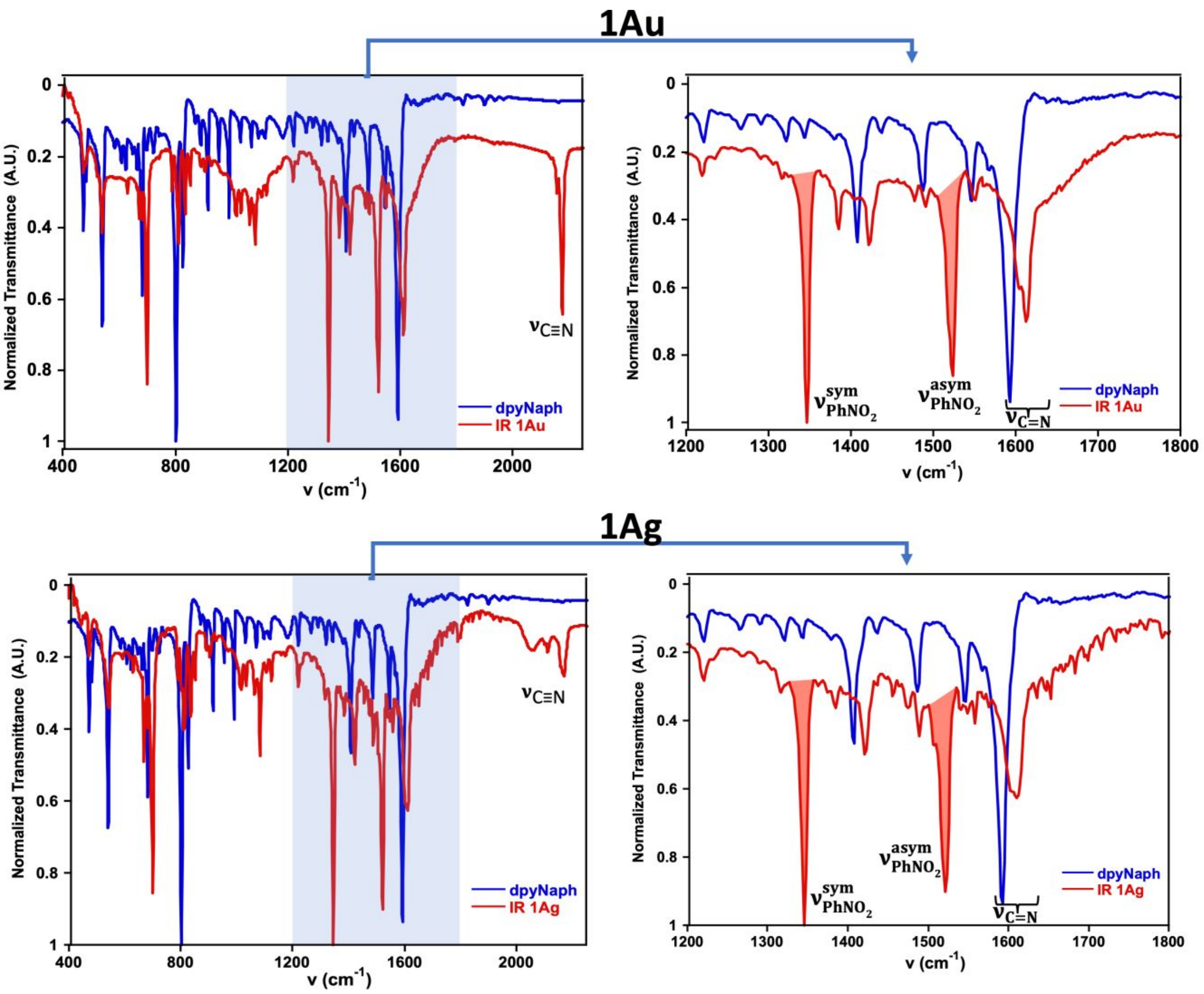

Table S1. Single crystal crystallographic data for **1Au** at 120 and 315 K.

**Temperature**	**120 K**	**315 K**
Empirical formula	$C_{36}H_{24}N_8O_4Au_2Fe$	
*M*r	1082.41	
Crystal system	triclinic	
Space group	P-1	
a (Å)	10.0700(2)	10.3885(3)
b (Å)	10.0964(2)	10.4780(2)
c (Å)	17.6195(3)	17.9800(5)
α (°)	95.407(2)	93.761(2)
β (°)	93.193(2)	92.095(2)
γ (°)	99.854(2)	106.424(2)
V ($Å^3$)	1752.39(6)	1870.13(9)
Z	2	
T (K)	120	315
D_c (mg cm^{-3})	2.051	1.922
F(000)	1024	
μ (Mo-K_α) (mm^{-1})	19.134	17.930
Crystal size (mm)	0.10x0.10x0.10	
No. of total reflections	7111	7549
No. of reflections [$I>2\sigma(I)$]	6115	5688
R [$I>2\sigma(I)$]	0.0295	0.0648
wR [$I>2\sigma(I)$]	0.0767	0.1860
S	1.056	1.098

$R_1 = \Sigma ||Fo| - |Fc|| / \Sigma |Fo|$; $wR = [\Sigma [w(Fo^2 - Fc^2)^2] / \Sigma [w(Fo^2)^2]]^{1/2}$.
$w = 1/ [\sigma^2(Fo^2) + (m P)^2 + n P]$ where $P = (Fo^2 + 2Fc^2) / 3$;
m = 0.0474 (**120 K**), 0.1521 (**315 K**);
n = 2.8490 (**120 K**), 2.3552 (**315 K**)

Figure S3. Overlay of the $[FeN_6]$ octahedrons in the LS (blue, 120 K) and HS (red, 315 K) emphasizing the small angular distortion induced by the change of spin state.

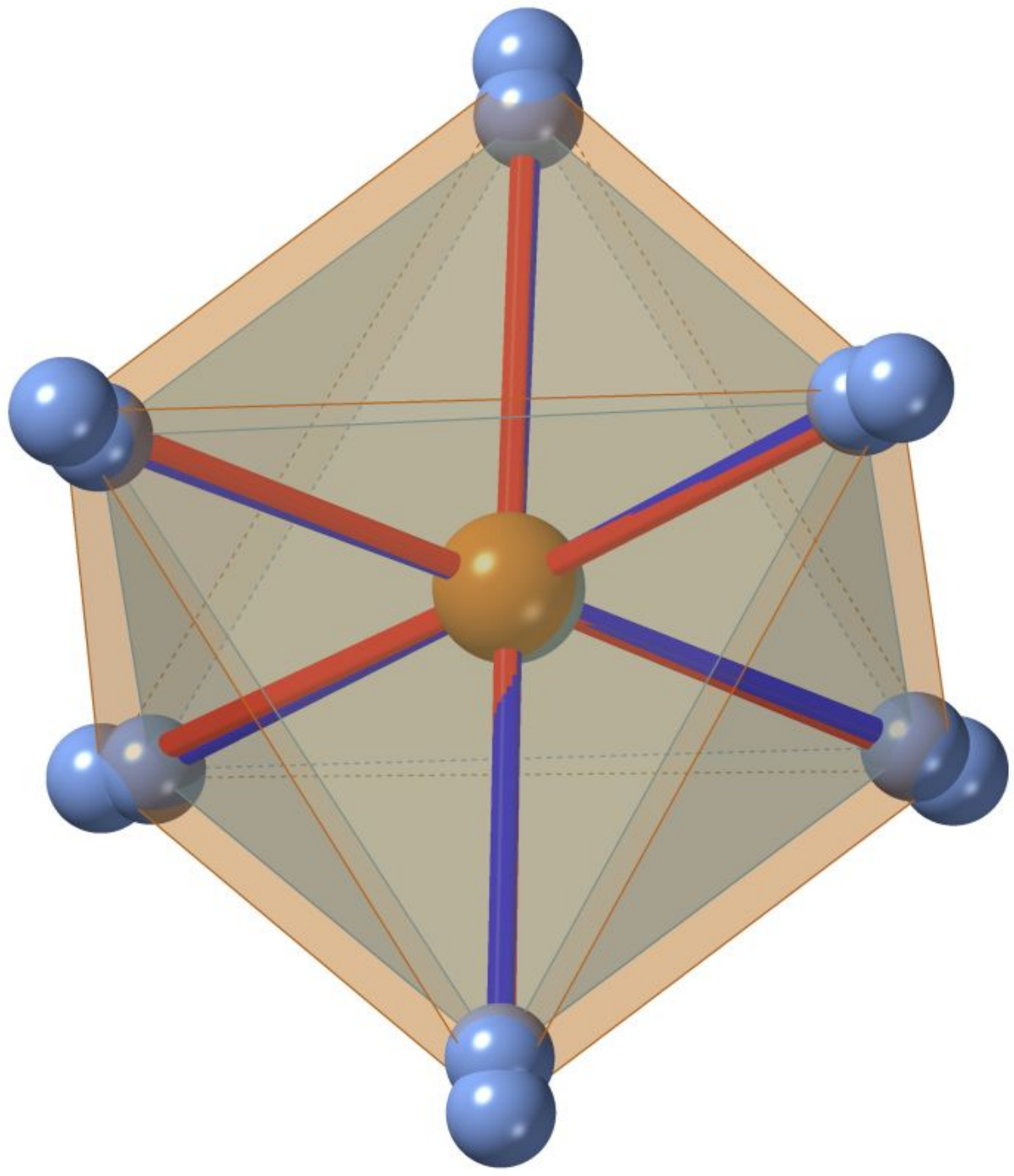

Table S2. Short intermolecular contacts smaller than the sum of the Van der Waals radii (VdW) for **1Au** at 120 K.

**Contact**	**Length**	**Length-VdW**	**Symm. Op.**
C3···H29	2.723	-0.177	-1+x,y,z; -1+x,y,z
C8···O2	3.139	-0.081	x,y,z; x,1+y,z
H8···O2	2.554	-0.166	x,y,z; x,1+y,z
C16···C26	3.304	-0.096	x,y,z; 2-x,-y,1-z
H6···O4	2.531	-0.189	x,y,z; x,-1+y,z
H11···O4	2.701	-0.019	x,y,z; x,-1+y,z
H16···O3	2.694	-0.026	x,y,z; x,-1+y,z
C13···C36	3.363	-0.037	x,y,z; 1-x,1-y,1-z
H26···H26	2.292	-0.108	-1+x,y,z; 1-x,-y,1-z
N7···O4	2.949	-0.121	-1+x,y,z; -1+x,-1+y,z
H36···H36	2.204	-0.196	x,y,z; 1-x,1-y,1-z

Figure S4. Intermolecular contacts shorter than the sum of the Van der Waals radii (VDW) for **1Au** at 120 K. Bicolor rods display the contacts indicated in **Table S2**.

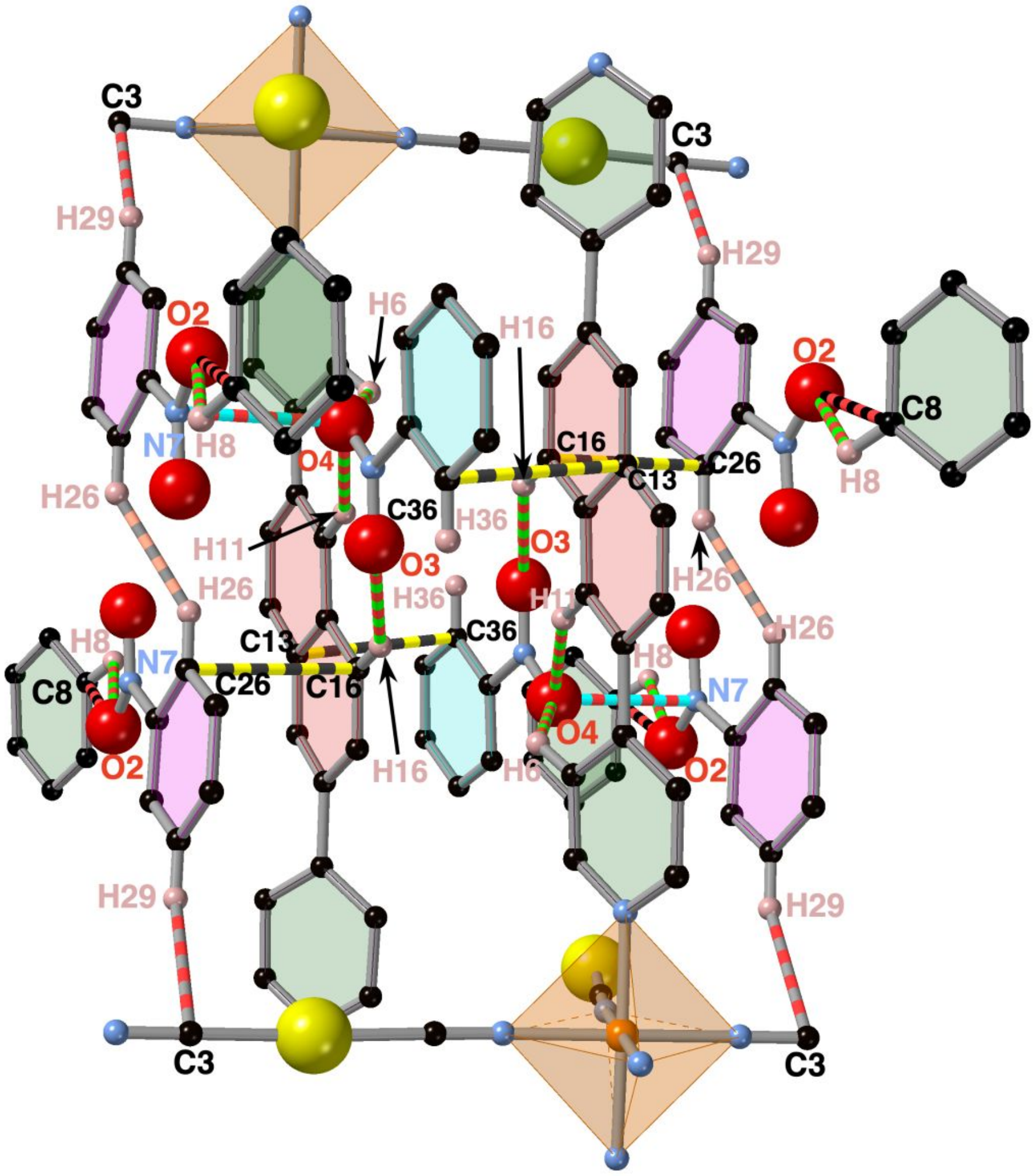

Table S3. Short intermolecular contacts smaller than the sum of the Van der Waals radii (VdW) for **1Au** at 315 K.

Contact	Length	Length-VdW	Symm. Op.
C8⋯O2	3.178	-0.042	-1+x,y,z; -1+x,1+y,z
H8⋯O2	2.589	-0.131	-1+x,y,z; -1+x,1+y,z
C6⋯O4	3.093	-0127	1+x,y,z; -1+x,-1+y,z
H6⋯O4	2.332	-0.388	-1+x,y,z; -1+x,-1+y,z
H11⋯O4	2.439	-0.281	-1+x,y,z; -1+x,-1+y,z
H16⋯O3	2.605	-0.115	-1+x,y,z, -1+x,-1+y,z
H26⋯ H26	2.327	-0.073	-1+x,y,z; 2-x,1-y,1-z
H36⋯H36	2.242	-0.158	-1+x,-1+y,z; 1-x,1-y,1-z

Figure S5. Intermolecular contacts shorter than the sum of the Van der Waals radii (VDW) for **1Au** at 315 K. Bicolor rods display the contacts indicated in **Table S3**.

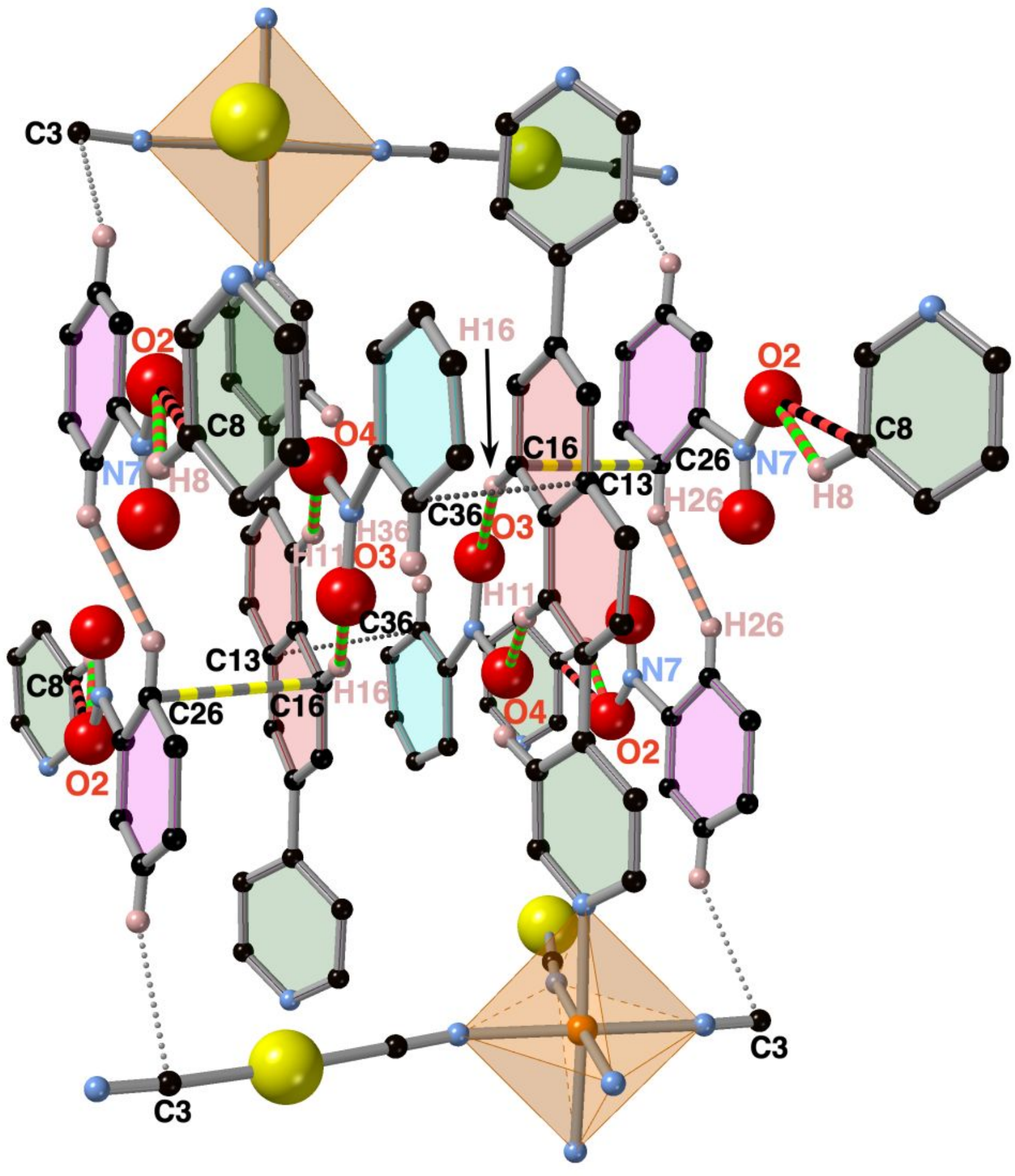

Figure S6. Intensity average lifetime as a function of temperature for **1Au**.

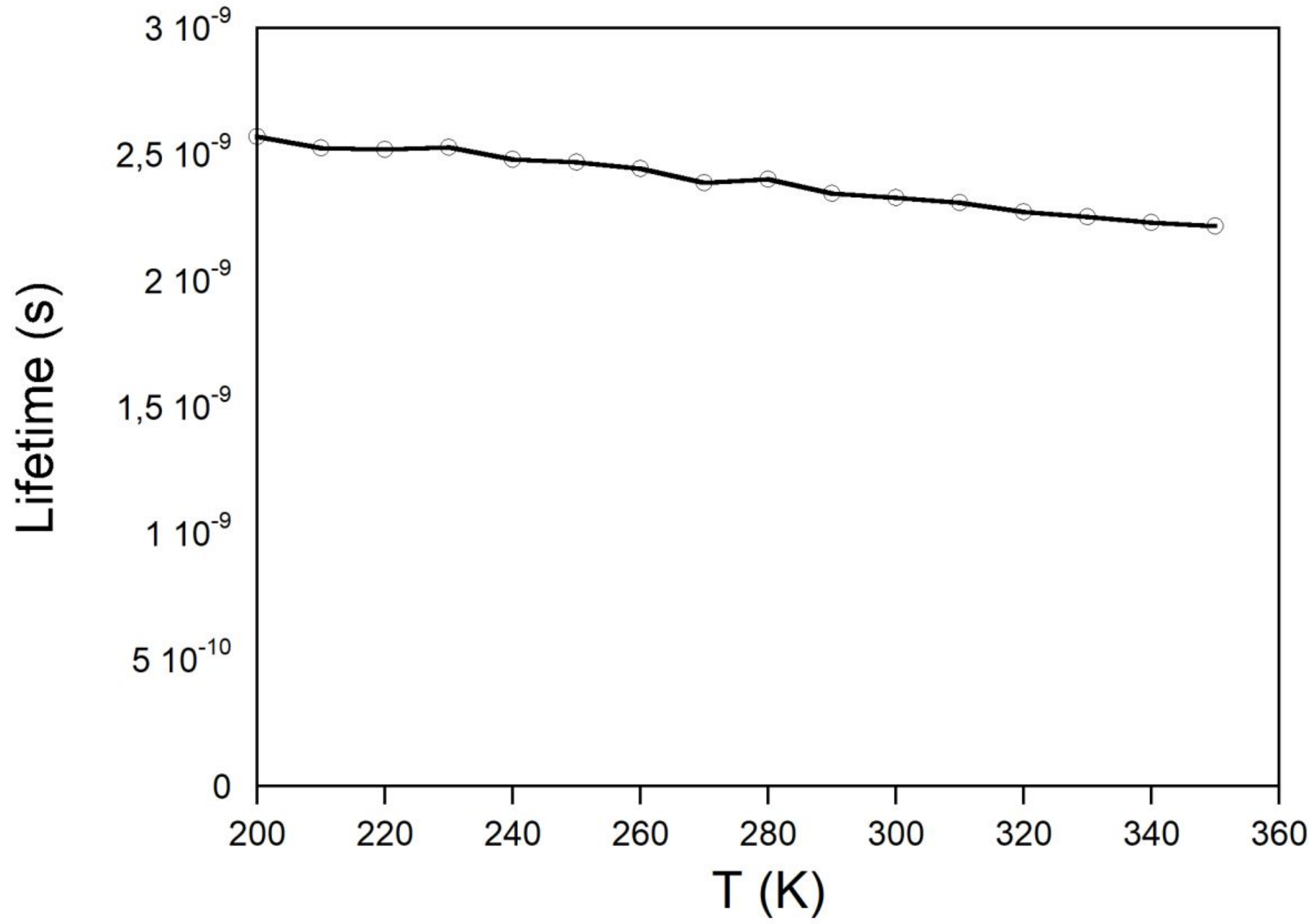

Figure S7. Thermal dependence of the fluorescence spectrum of the free ligand dpyNaph .

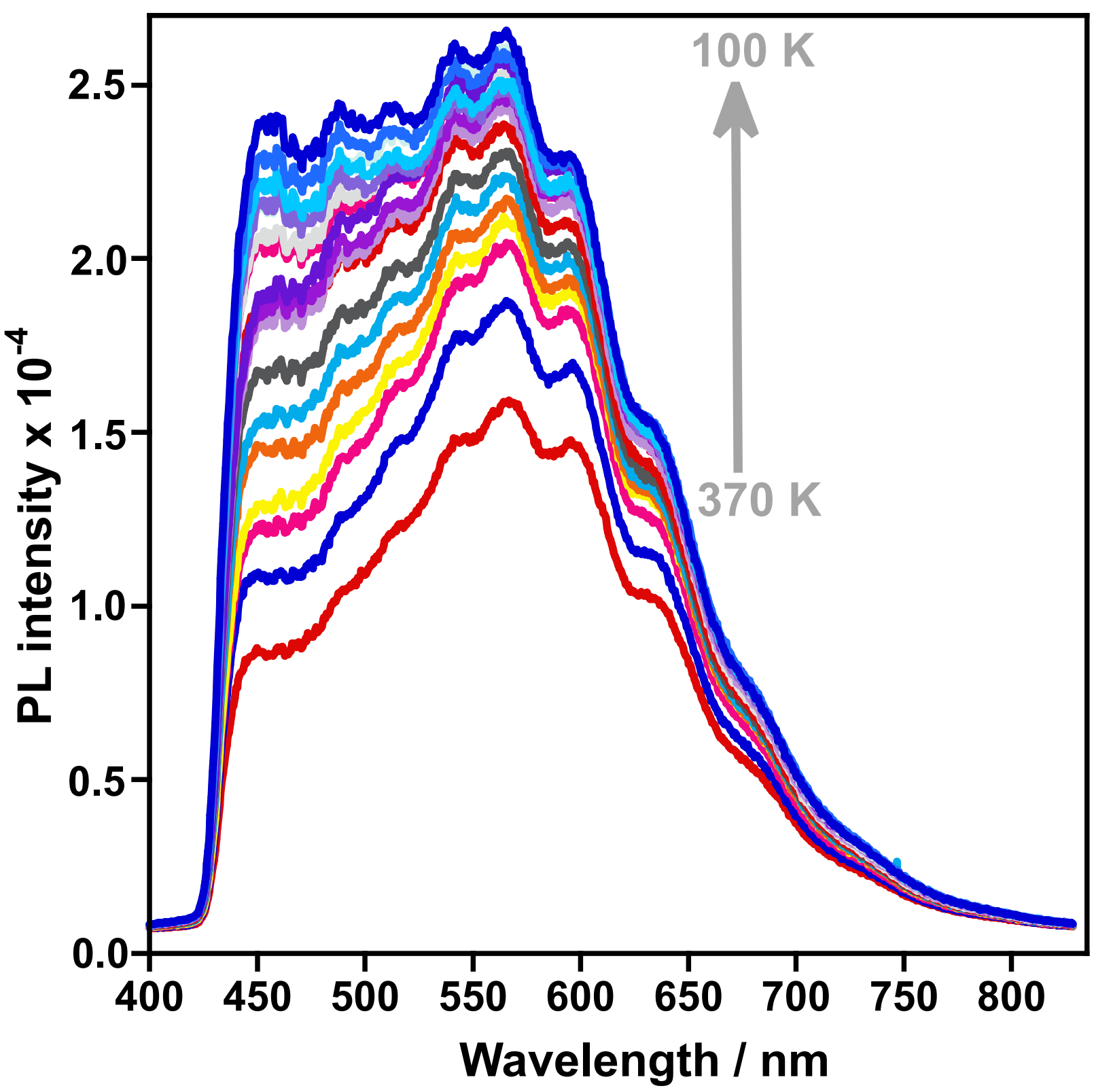

Figure S8. Comparison of the lowest 2θ diffraction peaks belonging to the [001] reflection for the title compounds **1Ag** (black line, RT) and **1Au** (red line, 120 K) compared with the homologous [100, 100 K] peak of the **3CHCl$_3$** solvate (see text).

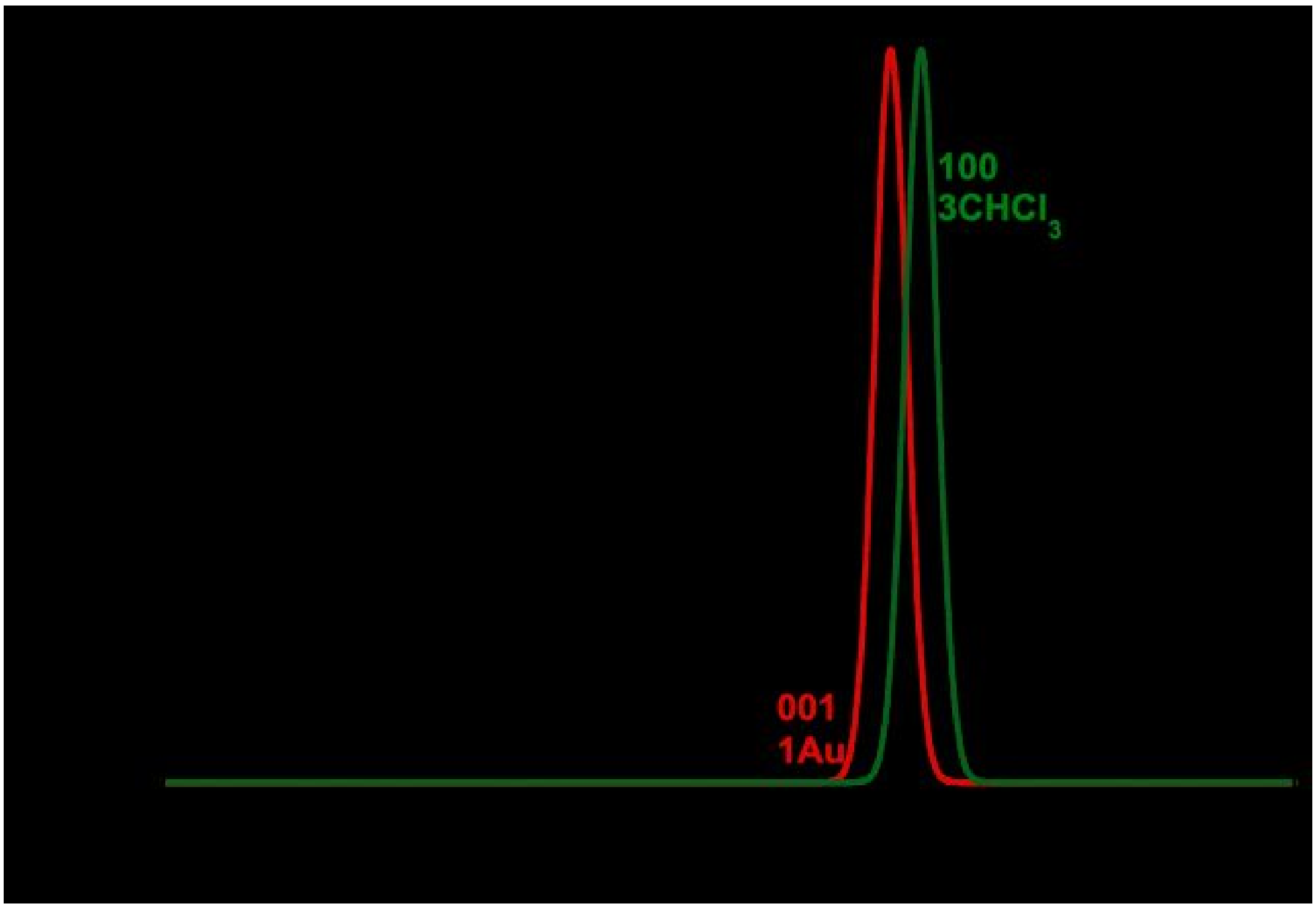

Supplement: Supplementary file 1 [file ic6c02744_si_001.pdf]
